# Early prediction of response to palliative chemotherapy in patients with stage-IV gastric and esophageal cancer

**DOI:** 10.1186/s12885-023-11422-z

**Published:** 2023-09-28

**Authors:** Xiaoyuan Ma, Eric Pierce, Harsh Anand, Natalie Aviles, Paul Kunk, Negin Alemazkoor

**Affiliations:** 1 Department of Statistics, University of Virginia, Charlottesville, USA; 2 School of Medicine, University of Virginia, Charlottesville, USA; 3 System and Information Engineering, University of Virginia, Charlottesville, USA; 4 Department of Sociology, University of Virginia, Charlottesville, USA

**Keywords:** Metastatic cancer, Esophageal and gastric cancer, Response prediction, Machine learning, Palliative chemotherapy

## Abstract

**Background:**

The goal of therapy for many patients with advanced stage malignancies, including those with metastatic gastric and esophageal cancers, is to extend overall survival while also maintaining quality of life. After weighing the risks and benefits of treatment with palliative chemotherapy (PC) with non-curative intent, many patients decide to pursue treatment. It is known that a subset of patients who are treated with PC experience significant side effects without clinically significant survival benefits from PC.

**Methods:**

We use data from 150 patients with stage-IV gastric and esophageal cancers to train machine learning models that predict whether a patient with stage-IV gastric or esophageal cancers would benefit from PC, in terms of increased survival duration, at very early stages of the treatment.

**Results:**

Our findings show that machine learning can predict with high accuracy whether a patient will benefit from PC at the time of diagnosis. More accurate predictions can be obtained after only two cycles of PC (i.e., about 4 weeks after diagnosis). The results from this study are promising with regard to potential improvements in quality of life for patients near the end of life and a potential overall survival benefit by optimizing systemic therapy earlier in the treatment course of patients.

## Introduction

It is established among medical professionals that what terminally ill patients need the most are: truth, touch, and time [1]. Specifically, they want their family and physicians to be truthful with them regarding their disease and its progress and treatments. Patients also want to be touched and be reminded that they are loved and valuable [1, 2]. Most importantly, patients wish to have more time. They need time to accept their illness and losses and resolve various issues arising from their upcoming death [1]. Maintaining quality of life as symptom free as possible is essential to optimizing their remaining time [3]. Treatment with palliative chemotherapy (PC) for patients with metastatic gastric and esophageal cancers has traditionally been the standard of care. The treatment paradigm also includes Her-2 targeted therapy and immunotherapy based on PDL1 status. PC has resulted in relatively modest survival benefits with the risk of significant side effects including but not limited to hair loss, extreme weakness, nausea, and mucositis. Currently, there is no biomarker or other predictor for responses to PC in patients with gastric and esophageal cancers [4]. In other words, only time shows whether PC is beneficial or not. In fact, the most common practice for evaluating the patients’ response to chemotherapy is to perform imaging after approximately two to three months of the treatment. Signs of clinical improvement in addition to imaging surveillance are also used to assess for evidence of treatment response. Clinical signs of treatment benefit such as improved appetite, weight gain, improved energy, and less dysphagia are monitored. Although these signs are correlated with effectiveness of PC, one cannot guarantee the improved clinical signs will result in an overall survival improvement. Unfortunately, about $30\%$ of the patients do not live more than three months after the initial diagnosis [5–7]. Consequently, early prognosis prediction for them is highly critical. In fact, many of these patients suffer from the toxicity of PC through their final days. If these patients had known their outcome ahead of time, they could have instead tried a potentially beneficial second-line treatment. Alternatively, they may have simply chosen to terminate the treatment and spend their remaining time more peacefully. The fact that these patients do not have enough time remaining for a trial and error approach to treatment makes early prognosis prediction for them crucial.

Several studies have attempted to improve our understanding of the prognosis of gastric and esophageal cancer. These studies mainly examine the correlation between survival time and patient or tumor characteristics, including but not limited to age [8], HER2 overexpression [9], sex [10], tumor size [11], metastasis sites [12–14], and laboratory variables [15]. Although these studies offer insights on what factors may lead to poor prognosis, they do not provide *response predictions* that can be used by oncologists or patients to make the decision to cease or continue PC. Few studies have attempted survival prediction for gastric and esophageal cancers, let alone cancers at stage-IV. In this work, we investigate the use of machine learning to predict survival for patients with stage-IV gastric and esophageal cancers. Machine learning tools, because of their ability to capture complex non-linearity within data [16], have proven to be capable in cancer risk prediction [17], detection [18, 19], classification [20, 21], and prognosis prediction [22, 23]. Nevertheless, only handful of studies have leveraged machine learning to predict prognosis in gastric and esophageal cancers. These studies mainly predict long-term survival (i.e., 3 or 5-year survival) based on serum markers, immunomarkers and clinicopathological parameters [24–27]. In fact, the literature lacks studies focusing on patients with the metastatic disease even though prognosis prediction is more vital for these patients given their limited time. Moreover, none of the existing research examines the *response* to chemotherapy. Specifically, the available survival prediction models use the value of biomarkers at diagnosis time and do not consider their evolution after chemotherapy treatment.

This work addresses a major gap in the literature. It targets one of the most vulnerable groups of cancer patients and aims to improve the quality of their remaining life by predicting the response to chemotherapy and its benefit in terms of survival extension. To this end, we investigate the possibility of predicting survival at the time of diagnosis and after two cycles of PC treatment. To the authors’ best knowledge, this is the first attempt in the literature to predict survival based on blood biomarkers for stage IV-patients in early stages of treatment.

We find that, with the information on metastasis sites and blood biomarkers at the time of diagnosis, machine learning models can predict whether a patient would survive beyond 6 month or 9 months, if they go through PC, with more than 75% accuracy. The accuracy of prognosis predictions increases to more than 85% when updated blood markers after two cycles of PC are included in the prediction models. Our hope is not only to leverage information about the evolution of blood markers for making early decisions on continuing, altering, or ceasing PC for end-stage gastric and esophageal cancers, but also to provide a blueprint for similar investigations in other types of cancers.

## Methods

### Data description

**Fig. 1:**
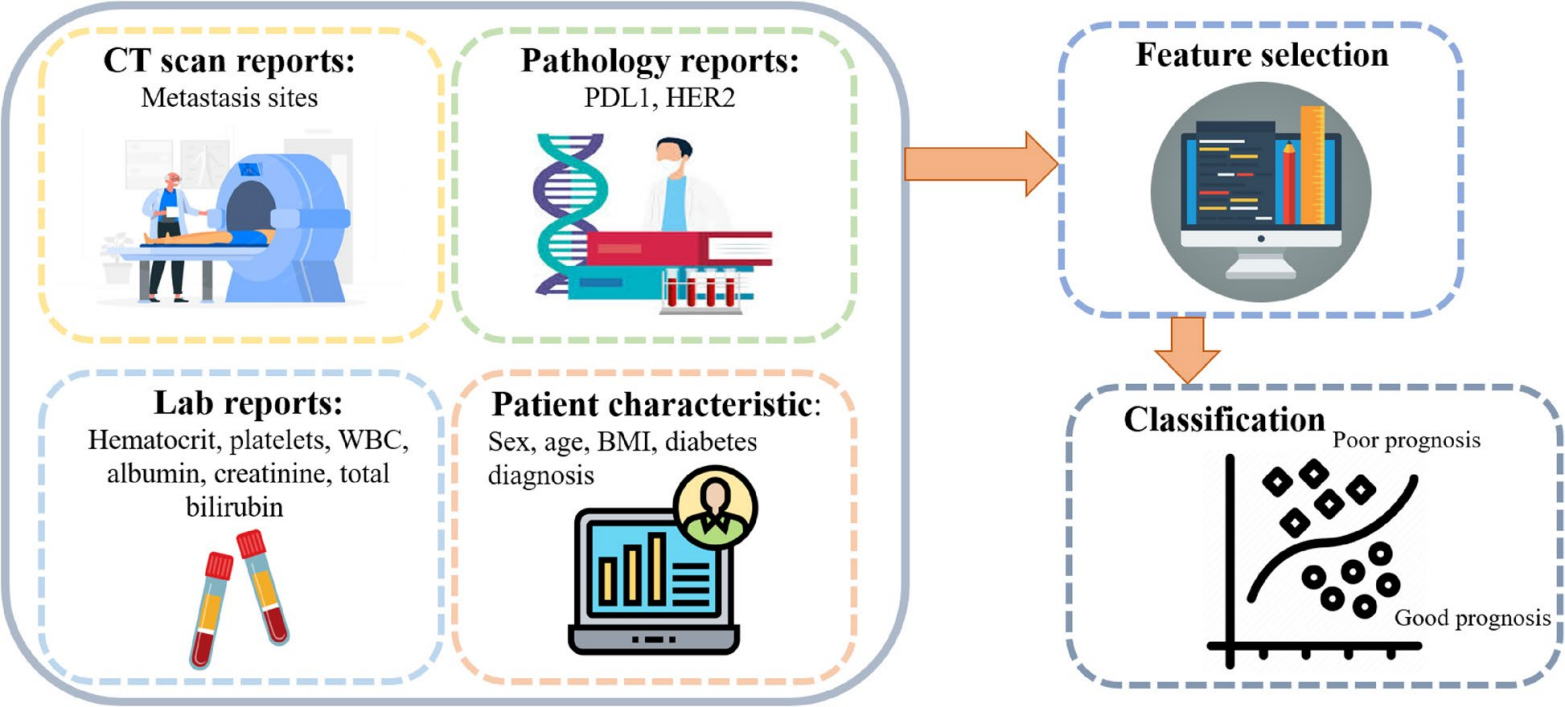
The schematic diagram of the data and framework used in this study

In this work, we used data from 150 patients with stage-IV gastric and esophageal cancers who were treated with standard PC at the University of Virginia Hospital over a ten year period from 2010 to 2020. For all patients, information on age, gender, body mass index (BMI), diabetes diagnosis, pathology and CT scan reports, as well as comprehensive metabolic panel and complete blood count panel tests (performed every two weeks before each chemotherapy treatment) are available. Specifically, the blood biomarkers included in this study are Hematocrit, albumin, platelets, WBC, creatinine, and total bilirubin. More specifically, we only included those blood biomarkers for which data for all patients was available. Other blood biomarkers such as CEA, CA19-9, and ctDNA were only available for small portion of patients and hence were excluded from the study. Additionally, pathology reports were searched to identify patients’ Her-2 and PDL1 status. Lastly, data for the primary tumor location and metastasis sites were extracted from CT scan reports. Specifically, we defined binary variables to indicate whether the primary tumor was located in the esophagus or stomach (and when the binary variable is one for both, it indicates the existence of the tumor in the junction). We also established four additional binary variables to determine whether cancer had spread to lymph nodes, liver, lungs, or unusual metastatic sites, which were included in the study. Notably, any metastatic site other than lymph nodes, liver, and lungs (e.g., brain or bone) was considered within the unusual metastatic site category. This categorization resulted from the relatively small number of patients with these uncommon metastatic sites, making it impractical to create separate variables for each one. Figure 1 shows a schematic view of different components of the dataset. The available bi-monthly lab data provides a unique opportunity to predict the prognosis given changes in blood markers after receiving PC for a short period. We have made the anonymous data set publicly available (see data availability statement), and interested readers can refer to it for more details about the data.

### Poor prognosis versus good prognosis

**Fig. 2:**
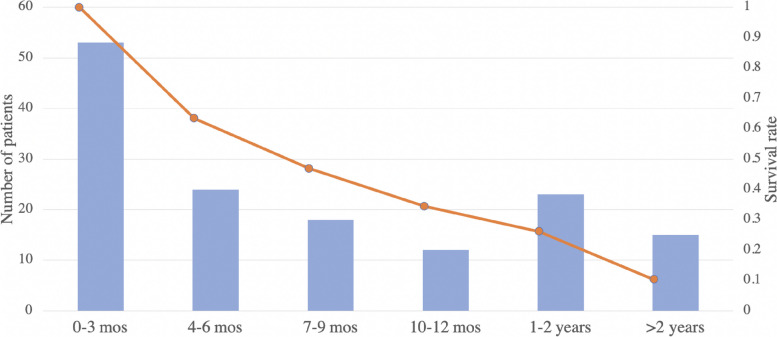
Overall survival of stage-IV gastric and esophageal cancer under PC treatment

The goal of therapy for the majority of Stage-IV gastric and esophageal cancer patients is non-curative. This raises the question of what should be considered as a good prognosis. Note that the expected overall survival for these patients is 4.3 months if they refuse PC treatment [28]. Figure 2 shows the distribution of overall survival of patients treated with PC. Even with PC treatment, a third of the patients will pass away during the first three months after diagnosis, half of patients die within six months, and only 37% patients survive beyond 9 months. We, therefore, developed different models that predict whether a patient would live beyond 6 or 9 months.

### Feature selection and prediction models

Cox proportional hazards regression (Cox model) is the most widely used approach for survival analysis in cancer research [29–31]. Cox model investigates the association between covariates and the survival time of patients. Despite its ability to identify factors that influence survival time, the Cox model cannot directly predict survival time since it is a hazard model (i.e., it is not a prediction model). We exploit the Cox model for feature selection. Specifically, we only pass those covariates that are found to impact survival time to the machine learning models which are then trained to predict prognosis. In particular, we investigated five widely used machine learning classifiers: Random Forest, Support Vector Machine, Quadratic Discriminant Analysis, Naive Bayes, and Neural Networks. All classifiers were trained and calibrated using cross-validation with 75% of the data. The classifiers were tested on the remaining 25% of the data and their accuracy is reported in terms of Area Under the Curve (AUC), which is widely used as the accuracy measure in cancer research or any type of classification problem in general [32, 33].

## Results

### Selected features

Tables 1 and 2 summarize the coefficients of Cox model for the selected features at the time of diagnosis and after two cycles of PC. Since the Cox model is a hazard model, an increment in covariates with positive coefficients is expected to increase hazard, thereby reducing survival time.

**Table 1:** Coefficients of Cox model at the time of diagnosis

Feature	Coef	z	p
Gender (0 for males, 1 for females)	0.399	1.894	0.058
Albumin at the time of diagnosis	-0.599	-3.648	<0.001
WBC at the time of diagnosis	0.076	3.094	0.002
With unusual metastasis sites	0.769	3.482	<0.001

**Table 2:** Coefficients of Cox model after two PC cycles

Feature	Coef	z	p
WBC after two PC cycles	0.093	4.13	<0.001
Albumin after two PC cycles	-1.035	-5.962	<0.001
Creatinine after two PC cycles	0.342	2.464	0.014
Total bilirubin after two PC cycles	0.098	1.895	0.058
With unusual metastasis sites	0.646	2.878	0.004

**Fig. 3:**
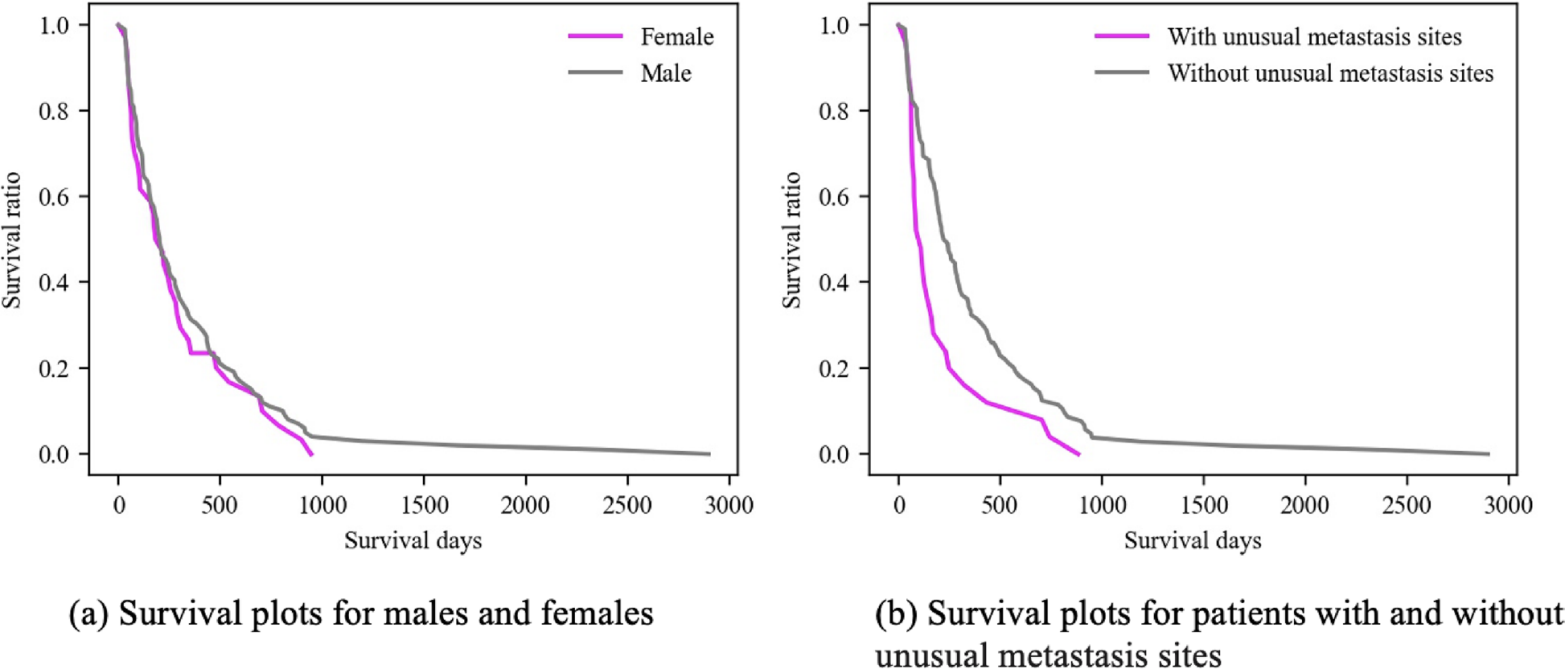
Survival plots of patients with stage-IV gastric and esophageal cancers categorized by gender and metastasis sites

One interesting observation is that the location of the primary tumor is not among the variables that significantly impact survival. This finding indicates that patients with gastric and esophageal cancer have similar survival patterns, which are primarily determined by the value of blood biomarkers (that can be influenced by the location of metastasis sites). To provide an insight into the survival impact of each of the two binary covariates selected by the Cox model, Fig. 3 shows the survival plots for patients grouped on these binary variables. Specifically, Fig. 3a shows survival plots for males and females, with male patients having slightly better survival. The observed slight difference, however, can be a result of small data size and may not be a general trend. Moreover, gender is not selected by the Cox model as a significant feature when blood markers after two PC cycles are included in the model. In other words, it is possible that the lack of enough significant variables in the initial Cox model has led to selection of Gender in the model. On the other hand, as shown in Fig. 3b, the survival plots for patients with and without unusual metastasis sites are significantly different. This is intuitive and in line with the literature.

**Fig. 4:**
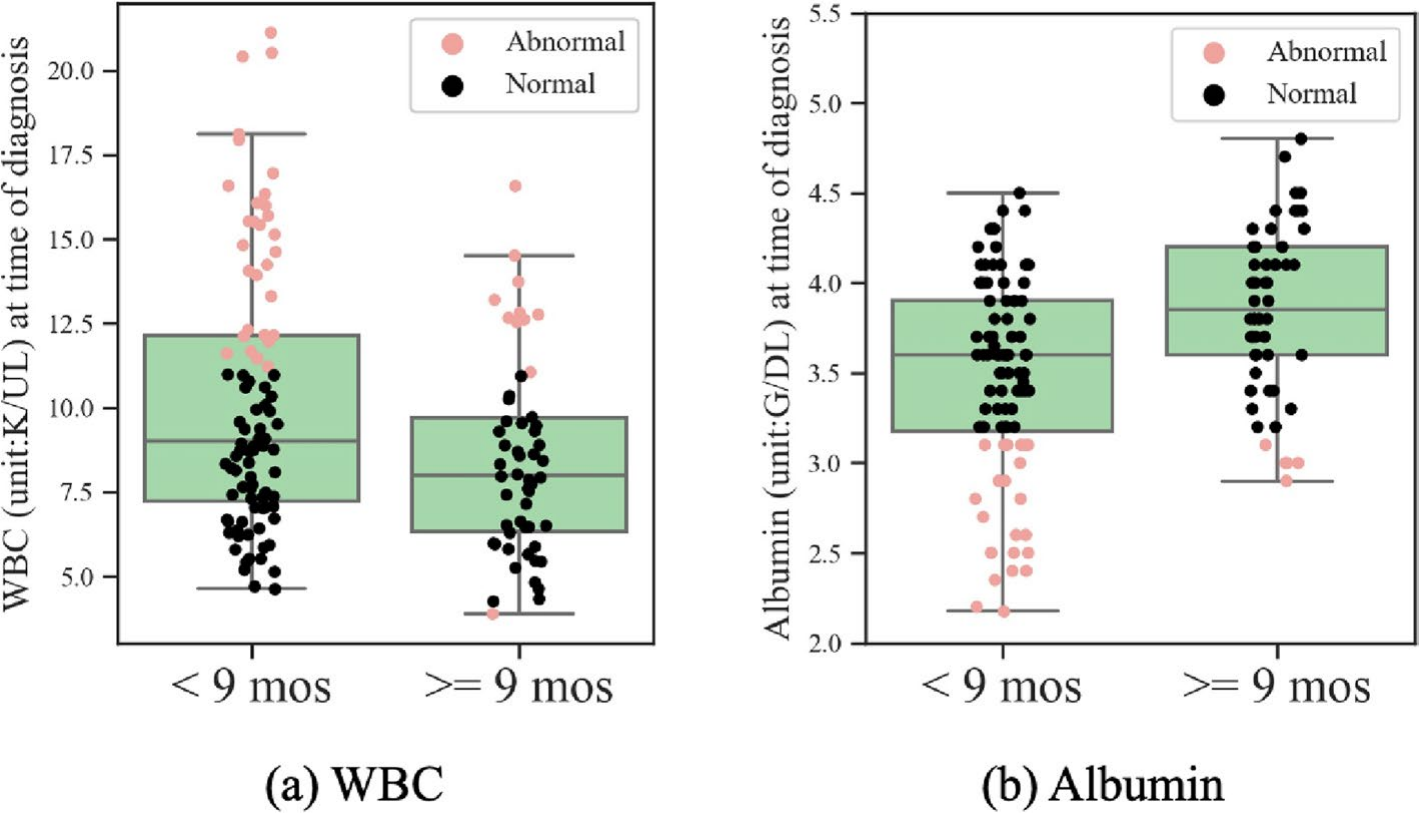
Distribution of WBC and Albumin for patients with poor and good prognoses at the time of diagnosis

**Fig. 5:**
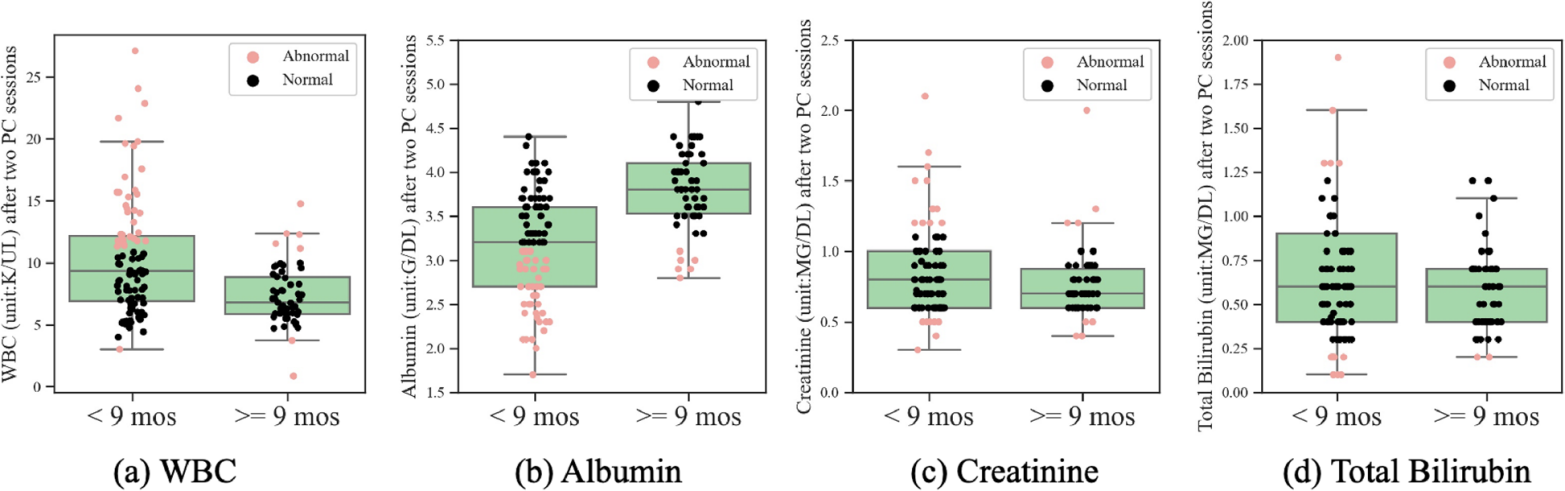
Distribution of blood markers for patients with poor and good prognoses after two PC cycles

Figure 4 depicts the box plots of WBC and albumin, the selected blood markers in the Cox model at the time of diagnosis, for patients with good and poor prognosis, where good prognosis indicates surviving longer than 9 months. Figure 5 depicts the box plots of WBC, albumin, creatinine and total bilirubin, the selected blood markers in the Cox model after 2 PC cycles, for patients with good and poor prognosis. Box plots show “minimum”, first quartile, median, third quartile, and “maximum” of blood markers in each group, where the “minimum” and “maximum” are defined as first quartile minus the 1.5 interquartile range and third quartile plus 1.5 interquartile range, respectively. Individual values of data are also shown along the box plots to provide an insight on the percentage of patients with normal and abnormal blood markers within the two groups with poor and good prognoses. It can be seen that the distribution of different blood markers is substantially different between patients with good and poor prognoses. Specifically, it is observed that larger percentages of those patients that survive less than 9 months have abnormal blood markers. For example, at the time of diagnosis, 32% of those with poor prognosis have abnormal Albumin while only 13% of patients with good prognosis have abnormal Albumin. Interestingly, it is observed that this difference becomes even larger after two PC cycles. Specifically, after two PC cycles, 52% and 11% of patients with poor and good prognoses have abnormal Albumin, respectively. Similar trend is observed for WBC. At the time of diagnosis, 32% of those with poor prognosis have abnormal WBC while only 18% of patients with good prognosis have abnormal WBC. This difference increases after two PC cycles leading to 37% and 12% of patients with poor and good prognoses having abnormal WBC, respectively.

### Prediction accuracy

**Fig. 6:**
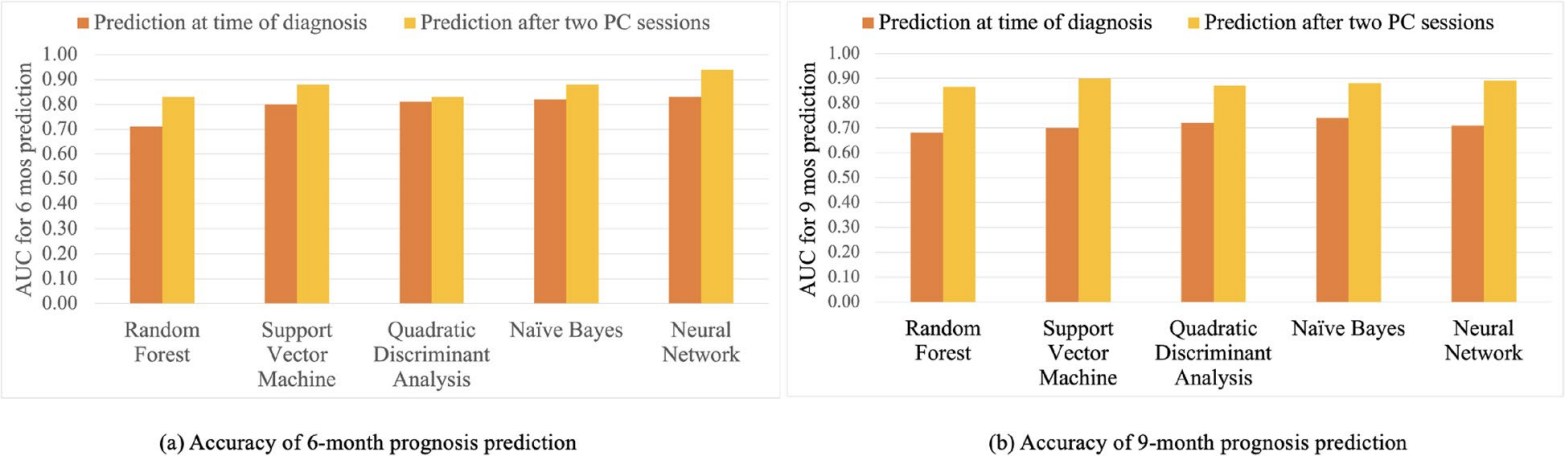
Accuracy of 6/9-month survival predictions at the time of diagnosis and after 2 PC cycles

Looking at Figs. 4 and 5, it is clear that there is not a single blood marker that determines the prognosis outcome. We used machine learning approaches to investigate the possibility of predicting prognosis based on the collective information provided by different blood markers. Specifically, the machine learning models predict whether a patient would live beyond 6 or 9 months. Figure 6 shows the accuracy of different classifiers for prognosis prediction. For 6 month prognosis prediction, it can be seen that, even at the time of diagnosis, most machine learning models provide more than 80% accuracy. Once the values of blood markers after 2 PC cycles are included, the accuracy increases to more than 85%. Specifically, it is observed that, regardless of the type of machine learning algorithm that is used, prediction accuracy is improved with the inclusion of the values of blood markers after 2 PC cycles. Such improvement is even greater for the 9 month prognosis predictions. Overall, the results show that it is possible to predict, with accuracy above 85%, whether a patient would live beyond 6 or 9 months at very early stages of the treatment. This paves the way for more personalized treatments of end-stage cancer patients, where no patients would suffer the demon of PC when it comes with no benefit.

## Discussion

Gastric and esophageal cancers are the fifth and eighth most common cancers worldwide [34, 35]. The prognosis of these cancers are favorable only in the early stages. However, more than a third of patients are diagnosed with stage-IV disease [5] as these cancers are mostly asymptomatic during the early stages. Consequently, the overall 5-year observed survival rate for patients diagnosed with gastric and esophageal cancers are among the lowest for all cancers. Specifically, the 5-year survival rate for metastatic gastric and esophageal cancer is less than 5% [5, 6].

Considering the short life expectancy of stage-IV patients with gastric and esophageal cancers, PC is established as the standard first-line treatment. PC is associated with modest survival benefits and patient quality-of-life improvement [36]. However, the response to PC varies significantly among the patients. This means that not only are there patients that do not necessarily benefit from PC but also their quality of life deteriorates significantly because of the substantial and cumulative side effects of chemotherapy [37]. It is estimated that between 20% to 50% of terminally ill cancer patients undergo chemotherapy in the last thirty days of their lives without any clear benefits, and in many cases experience significant toxicities, financial costs, and decreased quality of life [38]. Moreover, receiving PC has been shown to be associated with higher rates of cardiopulmonary resuscitation and mechanical ventilation in the last week of life. Also, patients receiving PC were more likely to die in an intensive care unit rather than in their preferred place [39]. End-stage patients with gastric and esophageal cancers can, therefore, tremendously benefit from early predictions of their prognosis. Such a prediction can help the patients and their physicians in the timely termination of PC when it is not beneficial, thereby improving the patients’ end-of-life quality. Patients and their care team may also leverage early prediction of response to PC to switch to a possibly more effective second-line treatment.

Unfortunately, despite the significance of implications of a reliable and easy-to-use response prediction model for patients with metastatic disease, few studies address this topic. First, the majority of available work concerning survival analysis investigates only the correlation between survival and potential biomarkers [8–10, 12–15, 40], without providing rigorous survival predictions. Second, studies that do attempt to predict survival do not focus on patients with metastatic disease [24–27]. The accuracy of a prediction model developed using data from patients in different stages are likely not sufficient for end-stage patients with shorter survival time. Moreover, none of the existing works has investigated the advantages of incorporating the changes in blood markers after a few cycles of PC in predicting the response to PC.

This work marks the first study predicting the response to PC for patients with metastatic gastric and esophageal cancer. Unique to this study, we use data from a comprehensive metabolic panel and complete blood count tests after two cycles of chemotherapy. We find that blood markers, including WBC, Albumin, Creatinine, and total Bilirubin after two cycles of chemotherapy are among predictors of prognosis. Different machine learning tools are able to provide highly accurate predictions (with AUC above 85% shown in Fig. 6) given the same predictors. The consistency of results among different machine learning models adds confidence in the true predictive ability of the input variables.

One primary advantage of the developed prediction model is its dependence solely on the metastasis sites and blood markers. Blood laboratory tests are performed regularly for patients undergoing chemotherapy (e.g., normally before each session of chemotherapy). The blood markers are, therefore, easily accessible and can be readily used in prediction models. Recently, estimating survival based on radiomic features from CT scans or tumor genomic profiles has garnered more attention for more common types of cancers (e.g., lung and breast cancer) [41–45]. This is despite the fact that both radiomic features and tumor genomics are not readily available. Specifically, extracting radiomic features (which contain distinct attributes associated with attenuation, shape, size, location, intensity, and texture of tumors) include two non-trivial steps: 1) segmenting the region of interest in CT images using manually delineated contours [46] or (semi) automatic packages such as 3DSlicer [47], 2) passing region of interest into software programs (e.g., TexRAD [48] and MaZda [49]) that extract the desired features. As for tumor genomic profile, the cost of tumor genomic profiling can be up to $100,000 per patient [50]. Consequently, specialized expertise and high monetary cost prohibit the wide utilization of prediction models based on radiomic or genomic features. This further highlights the importance of developing prediction models that are practical, given the availability and cost of existing technologies. Nevertheless, future research could explore the possibility of improving the prediction accuracy using radiomic or genomic features, in hope that extracting these features becomes less cumbersome and costly with advancements in technology.

We also want to call attention to the fact that it is very hard and time-consuming to obtain cancer patient data. There is limited availability of public sources for detailed cancer patient data (i.e., data with information beyond gender, sex, and age of patients). In fact, to author’s best knowledge there is no public dataset that includes the gastric or esophageal patients’ blood biomarker during their course of treatment. For this reason, the majority of studies in the literature that are focused on survival analysis or prediction use data from a specific hospital, which limits the sample size. The small sample size excludes the potential use of some machine learning algorithms (such as deep learning) that require extensive training with large datasets. Moreover, the private status of the data makes the validation or replication of the results impossible for other researchers. As a step forward to address this issue, we share the anonymous data used in this work on GitHub (see data availability statement) and encourage other researchers in the field to do the same in order to maximize the utility of the conducted research. Integration of datasets from various hospitals would provide innumerable opportunities to substantially advance the state-of-art in the field of cancer prognosis prediction.

Lastly, it must be noted that the data set used in this study lacked information about details of chemoteraphy regimens. It might be possible to further improve the results by considering this additional information. Moreover, relatively recent evidence supports standard first-line treatment for stage IV esophageal and gastric cancer as combined chemotherapy and immunotherapy in subsets of patients based upon PDL1 status. Currently, we do not have enough data to evaluate the performance of predictions models in predicting survival for patients receiving chemoimmunotherapy. Nevertheless, our results show that it is possible to predict how long a patient would survive at very early stages of treatment. As a future research path, we will study data from various institutions and will develop prediction models for different treatments (i.e., PC +/- immunotherapy). Such models built on large multi-intuitions data for different types of treatment can tremendously help patients and their care team to make an informed decision about their choice of treatment, and its continuation or termination.

## Data Availability

The raw data (including pathology and radiology reports and bi-weekly labratory results) is not publicly available. However, the cleaned and processed dataset used in this study is available on Github: github.com/Elsiexiaoyuan/gastric_esophageal_cancer_data. The code is available upon request.
